# The Neuroscience of Face Processing and Identification in Eyewitnesses and Offenders

**DOI:** 10.3389/fnbeh.2013.00189

**Published:** 2013-12-06

**Authors:** Nicole-Simone Werner, Sina Kühnel, Hans J. Markowitsch

**Affiliations:** ^1^Physiological Psychology, University of Bielefeld, Bielefeld, Germany

**Keywords:** eyewitness memory, eyewitness testimony, face processing, offender’s memory, identification, fMRI, real-life events, brain imaging

## Abstract

Humans are experts in face perception. We are better able to distinguish between the differences of faces and their components than between any other kind of objects. Several studies investigating the underlying neural networks provided evidence for deviated face processing in criminal individuals, although results are often confounded by accompanying mental or addiction disorders. On the other hand, face processing in non-criminal healthy persons can be of high juridical interest in cases of witnessing a felony and afterward identifying a culprit. Memory and therefore recognition of a person can be affected by many parameters and thus become distorted. But also face processing itself is modulated by different factors like facial characteristics, degree of familiarity, and emotional relation. These factors make the comparison of different cases, as well as the transfer of laboratory results to real live settings very challenging. Several neuroimaging studies have been published in recent years and some progress was made connecting certain brain activation patterns with the correct recognition of an individual. However, there is still a long way to go before brain imaging can make a reliable contribution to court procedures.

## Introduction

Our face is a very salient part of our body and maybe the most memorable feature (Henke et al., 1994). We are able to remember a great amount of individual faces. Our sensitivity for the small differences between facial characteristics is higher than for any other object category (O’Toole, 2005). This is one of the reasons why face recognition does play such a fundamental role for identification of a culprit. This applies a fortiori, as faces are less alterable than clothes or even hair styles.

Since eyewitnesses yield crucial and sometimes even the only available evidence in court, their testimonies are of critical importance for the juridical system. Unfortunately, eyewitnesses’ memories are not immune to decay and distortions. In an investigation of several cases in which convicts had been exonerated by DNA evidence afterward, the National Institute of Justice asserted that mainly eyewitness testimonies had been the most compelling evidence (Connors et al., 1996). Although in some cases eyewitnesses might have deceitful intentions and even give false confessions (e.g., to protect themselves or others), false information is often provided inadvertently for reasons of memory distortion. Our memories can vanish partly or completely, can be deformed in many ways or entirely originate from illusion (Markowitsch and Staniloiu, 2012). But not only eyewitnesses’ memories are prone to distortion. Offenders’ memories can be modified by different factors, as well. Although in most cases face identification made by the offender is of less importance, it can become relevant given that eyewitnesses might misidentify a person as a culprit or in some cases might have deceitful intentions. For those reasons, memories of an offender are fundamental for his or hers defense. Furthermore, some evidence concerning deviated face processing in offenders could be obtained in recent years, concluding that faces are processed differently even during non-criminal events, as well as facial expressions (Pardini and Phillips, 2010). A better comprehension of altered brain activity in culprits might give an expedient contribution to the understanding of delinquent behavior.

For literature search and selection, we started with standard databases like PubMed, PsycInfo, and Web of Science and used a large quantity of different key words in order to receive the relevant literature for this article. The most fruitful ones have been amongst others: *eyewitness, face processing, face recognition, functional magnetic resonance imaging (fMRI), identification, offender*, and different combinations of these. Secondly, we also used the “related article” and “cited by” search. We decided not to restrict ourselves too strongly by this common approach and included some articles which were obtained differently but contributed significantly to our topic as well as a few book chapters. Selection criteria were currentness of data, meeting the methodological demands, and topic relatedness. For other domains like memory influencing factors in the introduction, we conducted a separate literature search using other keywords, mainly *memory* in combination with different terms like *alcohol, age, amnesia, attention, context dependent, emotion, false memory, intoxication, stress*, and *trustworthiness*.

By reviewing the eminent literature we will (a) give an introduction to the general factors that can affect memory storage and retrieval in eyewitnesses and offenders, with an excursus on their treatment in police investigations; (b) comprise in particular which brain areas are involved in face recognition and how such correlates are influenced by internal and external factors; (c) explore the role of face processing, memory distortions, and deceit in offenders; (d) examine the comparability of data obtained in a laboratory environment to actual events and (e) conclude by discussing the relevance of these findings for juridical purposes, while providing a perspective on the challenges that still lie ahead.

### False memories

Next to incomplete memory encoding and memory loss, the creation of false memories poses a considerable challenge for lawyers and experts. The term “false memory” refers to the recollection of an event that has actually been experienced differently or even not at all (Kühnel and Markowitsch, 2009). While memory loss usually works for the benefit of the culprit, false memories can lead to wrong convictions and imprisonment of innocent people (Busey and Loftus, 2007). They are a normal everyday phenomenon, but have serious consequences in court. When talking about eyewitnesses in the following, we have to keep in mind that offenders are also eyewitnesses to a certain extent and many memory influencing factors apply for their memories as well. Brainerd and Reyna (2005) reported a case of a man who after a suggestive police interview accused himself of murdering is infant son. Later evidence given by experts who assumed that the injuries were not likely to be caused in the way the accused had described and recordings of the police interview led to the assumption that the confession was coerced. Though false memories appear regularly, certain conditions can enhance their occurrence. In police interviews, caution is indicated, as eyewitnesses can be very prone to suggestions (Loftus, 2011). This may be promoted by the mental or physical constitution of the eyewitness (for example high emotional state, stress, or sleep-deprivation) or social demands like the wish to help the police and to catch the culprit. Zhu et al. (2010) also reported individual differences in false memory concerning the exposure to misinformation. They found a negative correlation with intelligence, perception, memory, and face judgment abilities. Misinformation can be obtained by conversation with other eyewitnesses or by suggestive questions during the police interview. Suggestive questions can include false or at least new information for the eyewitness. They can also facilitate the creation of false memories without providing any new content, for example by presuming that the eyewitness must have observed something (Brainerd and Reyna, 2005). However, questioning cannot only provoke false memories, but it can also induce forgetting (Migueles and García-Bajos, 2007; Camp et al., 2012). Retrieval practice normally enhances recollection of the studied items, but it can have opposite effects on related but not recapitulated information. This phenomenon has been observed with respect to, for example, the memory of offender’s characteristics. A multitude of factors affects eyewitnesses’ memory, such as the wording of questions, post-event information, confidence malleability, attitudes and expectations, exposure time, presentation format, or alcohol intoxication (Kassin et al., 2001).

Though guidelines for the treatment of eyewitnesses do exist (Technical Working Group for Eyewitness Evidence, 2003), Wright (2007) gave voice to his concern that jurors might not be aware of the differences between presentation formats for offender identification (e.g., sequential and simultaneous lineups) and their reliability. Apparently, not only eyewitnesses and suspects have to be handled with care for preserving their memories, but also judges, advocates, and jurors must be sensitized to possible memory influencing factors.

### Influences on Memory Accuracy and Quantity

Memory performance can be influenced by many different factors. Below we highlight some of those memory enhancing or impairing variables, although we cannot assume the possibility of giving an exhausting overview.

Age is one important factor that affects memory of events. Infantile eyewitnesses pose a special challenge to investigators, inasmuch as they are more susceptible to suggestions and research approaches are limited for ethical reasons. In a group of children aged 5–12 years old, older children were more prone to connect gist-based information across several events than younger children (Odegard et al., 2009). Clifford et al. (2012), who compared 7- to 8-year-olds with a group of 13- to 14-year-olds, also found the younger children to be more vulnerable to the detrimental effects of time delay in an identification task. In a study conducted by Humphries et al. (2012), adults made more correct identifications in the sequential lineup video condition than 5- to 6-year-olds and 9- to 10-year-olds, but not in the simultaneous and in elimination lineups. During target-absent lineups, adults exhibited higher correct rejection rates than children, regardless of lineup condition. At the upper end of the age range, findings are quite mixed. Elderly persons (aged 57–73) tended to make more false alarms than younger adults (aged 19–27) in an experiment by García-Bajos et al. (2012). They also performed poorer in a recall task, but only regarding actions untypical for the event (i.e., a bank robbery, a video of which had been shown to the participants). Nevertheless, there was no difference in recall between groups concerning highly typical actions. Notwithstanding lower recall scores, elderly persons seem to be less prone to misinformation effects under special circumstances. In an experiment by West and Stone (2013) younger adults made more mistakes due to misinformation than older adults when learning occurred incidentally, but not when it was intended. Maybe less complete information processing in the older group might have worked in this case to their benefit. Given these results, it can be concluded, that although differences exist between older and younger adults’ memory abilities, they may be more or less pronounced depending on the exact investigated skills.

The capability of identifying unfamiliar faces varies largely over individuals, and we still do not completely understand the underlying mechanisms. Megreya and Bindemann (2013) investigated the relationship between identification abilities and personality and found higher identification skills for women who scored low on anxiety and tension and high on emotional stability. The impact of personality on eyewitness testimony is still unclear, but although findings are still somehow heterogeneous, our understanding of other modulating factors is much less limited.

Most studies dealing with the influences of alcohol on eyewitness memory report alcohol intoxication to cause reduced recall accuracy (van Oorsouw and Merckelbach, 2012) as well as impairment of conscious recollection (Ray and Bates, 2006). However, familiarity-based recollection seems to be not affected (Bisby et al., 2010). Regarding the recognition of perpetrators, these findings imply that identification ability in lineups or photospreads in which the culprit is present is apparently not impaired by alcohol consumption, although it comes to more false identifications in culprit-absent trials (Yuille and Tollestrup, 1990; Dysart et al., 2002). Read et al. (1992) found that alcohol reduced person identification in a consecutive study, but only when participants’ arousal was low. In contrast, Schreiber Compo et al. (2012) could not find a significant difference between the amount of accurate or inaccurate details (as well as “I don’t know” statements) reported by sober and intoxicated participants. However, under certain circumstances, alcohol might even benefit memory. In a study by Moulton et al. (2005), alcohol administered after learning facilitated recall of prose passages read before. Perhaps alcohol preserves recently acquired memories by suppressing cognitive interference with new material. The impact of alcohol on eyewitness testimony may therefore depend on the timing of consumption. Interestingly, numerous studies have been performed since many decades that revealed state-dependent recall under alcohol (e.g., Goodwin et al., 1969; Hoffman et al., 1997). Weingartner et al. (1976) for instance found that encoding under alcohol intoxication should be followed by retrieval under alcohol intoxication. If individuals are sober at retrieval while they had learned the words while intoxicated, they performed poorer than in the matched condition. In any case, alcohol intoxication in witnesses during the incident seems to be widespread. Astonishingly, only few general guidelines for interviewing intoxicated eyewitnesses exist (Evans et al., 2009b), so that sober and intoxicated eyewitness are often treated quite similarly (Palmer et al., 2013). Nevertheless, the results of an experiment conducted by Evans and Schreiber Compo (2010) are somehow more encouraging: even if participants did not consider the whole range of potential factors and their interactions, they were quite aware of possible derogating effects of alcohol on eyewitnesses’ cognitive abilities and these considerations modulated their verdicts. But for including such considerations, jurors must be informed about possible intoxications and according to Evans et al. (2009b), 71% of the investigators do not use an instrument for determining the breath alcohol content when intoxication is suspected. This illustrates the need for standardized guidelines.

Results concerning the impact of stress on eyewitness memory are diverse as well. Stress can enhance memory for central events, especially in comparison to peripheral events, if stimuli are encoded under arousal and stress is experienced shortly after encoding (McGaugh and Roozendaal, 2002; Echterhoff and Wolf, 2012; McGaugh, 2013). Cahill et al. (2003) found a beneficial effect of post-learning stress on emotionally arousing stimuli but not on neutral items. Other studies argue for more detrimental effects of stress. In two studies described by Wolf et al. (2004), cortisol administration had more detrimental effects on the retrieval of neutral autobiographic episodes, while it impaired retrieval of emotional words from a word list (cf. Kuhlmann et al., 2005; de Quervain et al., 2007). The way in which stress affects memory for emotional content, seems to depend on the kind of stimuli learned. Kuhlmann and Wolf (2006) observed stress-induced recall enhancement for emotional pictures and an impairment for neutral pictures while recognition remained unaffected (cf. Liu et al., 2008). Stress can also exacerbate the misinformation effect (Morgan et al., 2013) and stress during encoding can lead to a more liberal response bias (Qin et al., 2012). Participants completing a high-intensity physical-assault exercise before encoding showed impaired performance in recall and recognition. They were also less able to identify a target in a lineup (Valentine and Mesout, 2009; Hope et al., 2012). Andreano and Cahill (2006) found an inverted-U relationship between dose of endogenous stress hormones and memory in men but not in women. Hence, stress intensity might also contribute to enhancing and impairing effects of stress. Furthermore, stress also modulates the extent of involvement of several brain structures (Schwabe and Wolf, 2012). In summary, the effect of stress on memory depends on many parameters like gender, stress intensity, emotional valence, time between learning and retrieval, context (Schwabe et al., 2009), time of stress induction (Smeets et al., 2008; Wolf, 2012), stimuli material, and its relatedness to the stressor (Smeets et al., 2009).

Several studies have been conducted concerning context-dependent memory. Congruence between the context of a perceived event and the retrieval situation can enhance memory for this event (cf. Smith and Vela, 2001 for a review). Context can refer, e.g., to the physical environment (Godden and Baddeley, 1975) or to the physiological state like intoxication (Eich, 1980) but also to the experienced mood during learning phase and retrieval phase (Fitzgerald et al., 2011).

One phenomenon with impact on our attention and therefore on our memory is referred to as “weapon focus.” Negative arousing objects in a scene (e.g., weapons) can reduce recognizability of peripheral information (Easterbrook, 1959; Kensinger and Schacter, 2007; Kensinger et al., 2007; Waring and Kensinger, 2009). In general, memory for details of negative arousing stimuli seems to be enhanced compared to neutral objects (Kensinger et al., 2006). On the other hand, emotionally aversive stimuli can disrupt later recall of previously retrieved neutral information (Strange et al., 2003, 2010). Interestingly, Hurlemann et al. (2005) could not only demonstrate negative items to elicit retrograde amnesia for preceding neutral words, but also found positive stimuli to induce hypermnesia for previously recapitulated neutral items. They also observed emotionally arousing stimuli – negative items as well as positive items – to provoke anterograde amnesia. Thus, valence and arousal seem to make different contributions to memory, with valence determining enhancing or impairing effects on retrograde memory and arousal affecting anterograde memory (Hurlemann, 2006). But the experience of an aversive event cannot only induce amnesia, it can also lead to anxiety disorders like post-traumatic stress disorder (PTSD) (Mineka and Oehlberg, 2008).

The influences discussed above rather concern general memory abilities and possible influencing factors. Below we now will address the more specific field of face recognition and the underlying neuronal processes.

## Memory and Face Processing in Eyewitnesses

Because humans are better able to distinguish between faces than between objects of any other category, this expertise in face processing led to a wide discussion about the existence of specialized brain areas which are solely responsible for face processing (Sato and Yoshikawa, 2013). A lot of light was shed by the examination of patients with *prosopagnosia*, an inability of identifying previously known persons by watching their faces (Damasio et al., 1990; Minnebusch et al., 2009). Typically these patients remain capable of recognizing relatives and friends from voice, categorizing faces as faces, and are often even able to interpret facial expressions (Tranel et al., 1988).

We have to consider that face recognition involves a lot of different processes. In the eyewitness context, the aim is to distinguish between familiar und unfamiliar faces *(Have you seen this man before?)*, or even better: to ascertain a special identity of a person *(Is this the man who offended you?)*. But a lot of variables modulate the way in which faces are analyzed, like the individual components of the face (Itier et al., 2006), race (Phelps et al., 2000; Behrman and Davey, 2001; Golby et al., 2001; Johnson and Frederickson, 2005; Turk et al., 2005), sex (Lewin and Herlitz, 2002; Rahman et al., 2004; Hofmann et al., 2006; Rehnman and Herlitz, 2007; McBain et al., 2009), expressed mood (Kaufmann and Schweinberger, 2004; Schulte-Rüther et al., 2007), movement (Roark et al., 2003; Lander and Davies, 2007), and the eyewitness’ age (Memon et al., 2003a; Firestone et al., 2007). Likewise, hair does have an effect on eyewitness accuracy (Wright and Sladden, 2003; Frowd et al., 2012c), but is often excluded from face recognition studies which might be wise also in respect of delinquents often wearing headgears.

Face processing always seems to cause activation in the face fusiform regions, but encoding and recall of learned faces must utilize more extended networks (Elgar and Campbell, 2001; Steeves et al., 2006; Lee et al., 2012; Parvizi et al., 2012), like participation of the anterior temporal cortex including the anterior tip of the collateral sulcus (Nestor et al., 2011; Nasr and Tootell, 2012). Rossion et al. (2012) used intact and scrambled versions of object and face pictures to unravel the neural mechanisms behind face processing in an fMRI block design experiment. They identified several clusters: e.g., in the pulvinar, inferior occipital gyrus, posterior superior temporal gyrus, anterior infero-temporal cortex, and amygdala, all with a pronounced right lateralization. The middle fusiform gyrus distinguished best between faces and objects but because of its concomitant differentiation between the pictures of cars and scrambled cars it was also identified as the least face-selective region of the ones mentioned above.

Even the kind of familiarity we experience while watching a face, the way in which we became acquainted with someone, does play a role. The processing of famous faces in comparison to personally familiar faces involves different brain areas (Sugiura et al., 2011). Von Der Heide et al. (2013) included 25 fMRI studies in a meta-analysis dealing with famous faces and familiar faces stimuli and also conducted an own empirical fMRI experiment with picture stimuli of faces differing in famousness and personal relationship. Baseline images consisted of blurred and landmark pictures. The authors found higher left-lateralized anterior temporal lobe activations for familiar faces, while activation associated with novel individuals was evoked in the right anterior temporal lobe. Activation connected to personally familiar faces and famous faces partially overlapped, but famous faces activation exhibited a more ventral pattern. The study design is somehow problematic as experimental tasks differed between famous and familiar faces. For familiar faces, participants rated which of two presented known persons they feel closer to, while in the famous faces condition, participants had to complete a 0-back task indicating if two images of the same category (famous vs. non-famous) appeared in succession. But it can be concluded that any of the mentioned aspects above, like the individual characteristics of face components and their relation to each other, evoke slightly different combinations of neural collaboration. This is one of the reasons why clarifying the underlying neural mechanisms is so demanding. For a holistic understanding, face recognition has to be broken down to many single processes, which probably do not stand for themselves, but depend on each other.

From a criminological perspective, the most intriguing aspects are: (i) the distinction between familiar and unfamiliar (Shah et al., 2001) and (ii) the precise source, i.e., where, when, and under what circumstances someone has been seen before. The first issue was addressed by Gobbini and Haxby (2006) who compared the neural responses to known faces with activation corresponding to new faces in an event-related fMRI study. Familiarity of known faces was induced experimentally in order to avoid any biographical or emotional content. The authors found higher activation in the precuneus while watching familiar faces. Observing new faces led to higher responses in the fusiform gyrus and the amygdala. The authors suggested that this might reflect higher encoding effort (or increased attentional load) and an elevated guarding function, respectively. In a preceding fMRI study (Gobbini et al., 2004), similar results have been found with the amygdala showing lower activation during presentation of personal familiar faces compared to famous familiar faces and faces of strangers. New faces were associated with higher activation in the fusiform gyrus in contrast to famous familiar faces. But no difference between faces of strangers and faces of personally acquainted persons was detected in this region. Familiarity’s effect on this area does not seem to be linear. Gobbini and Haxby (2007) also proposed a new model for face recognition consisting of the three elemental parts “visual appearance,” “person knowledge” (e.g., information about traits, intentions, attitudes, biographical facts, and episodic memories), and the “emotional response.” These components may involve different brain structures and failure to access one of them could lead to impaired recognition. The emotional response to the face of an offender will obviously differ from the reaction to beloved relatives or friends and consequently alter brain activation. Unrestricted transferability from those findings to criminal contexts is indeed questionable. To untangle the underlying neural mechanisms involved in recognition of a culprit’s face, we have to investigate more realistic settings which exhibit criminal content. But before we have a closer look on studies facing this issue, we must keep in mind that several methods and approaches exist to confront eyewitnesses with suspects or rather to extract the wanted facial details from their memories: like lineups (Clark and Tunnicliff, 2001; Wells and Olson, 2003), showups, photospreads (Yarmey, 2004), mug shots, or facial-composite production (Wells and Hasel, 2007). It is rather obvious that these different procedures do not only lead to different results in memory accuracy, but will also affect the incidental brain activity. Furthermore, there is evidence that the amygdala is active toward attractiveness of a face, particularly its eyes (Demos et al., 2008) and that individuals – such as boys with conduct problems and callous-unemotional traits – may be unable to detect the emotional expression of a face due to amygdala hypoactivity (Jones et al., 2009). Adolphs et al. (1998) also found three subjects with complete bilateral amydala damage to judge faces as more trustworthy and approachable than healthy individuals. These findings are in accord with the results obtained by Winston et al. (2002) who could also show in an event-related fMRI study the bilateral amygdala to be involved in ratings of faces as untrustworthy, as well as the right insula, while activation in the right superior temporal sulcus was correlated with judgments as trustworthy.

Lefebvre et al. (2007) measured event-related brain potentials in culprit present and absent lineup tasks at different levels of time delay. Participants first watched four videos, all showing a mock burglary incident. They were also instructed to pretend that they had observed a real crime, for which they were the only witnesses. Although memory accuracy deteriorated over time, P300 remained a reliable predictor for correct identification. In culprit-absent lineups, P300 was reduced. It would indeed be desirable to extract information about the identity of a culprit even when the eyewitness is unaware of the correct features. Aspiring to make a contribution to this issue, Iiadaka et al. (2012) induced false memories in a face recognition fMRI experiment. The authors used morphed pictures of faces to evoke a false familiarity for lure faces. During the test phase, participants were confronted with old, new, and lure targets in a randomized order. Feelings of familiarity were correlated to activation in the orbital cortices, as well as to neural response in the left amygdala. Here, activity was highest for correct responses, lowest for incorrect answers regarding old and new items. Neural reaction to incorrect answers concerning lure items (i.e., stating a lure item as old) and therefore related to false memories fell somewhere in between. A participation in false memories could be unveiled in a region in the anterior cingulate cortex. In this area, activation was correlated with the difference in reaction times observed for lure items. Very demanding is the question what happens when we misinterpret a face as familiar while the accordant person is unknown to us. Do we only mix up similar characteristics of two faces as it would be true for the lure items? Moreover, incorrect answers to completely new faces could be just as illuminating. Unfortunately, although Iiadaka et al. (2012) recorded participants’ confidence ratings (indicating how sure they feel about their responses), the authors could not differentiate between certainty statements due to a lack of answers expressed with high confidence (cf. Risius et al., 2013).

We must bear in mind our objective is not only to help eyewitnesses remember correctly and avoid the creation of false memories, but that eyewitnesses can also be deceitful for a variety of reasons (e.g., to protect themselves or others). Bhatt et al. (2009) conducted an fMRI study in which participants were confronted with target present and target absent photo lineups. Targets had been learned previously. The subjects were partly instructed to lie and to conceal the identity of the learned face and to pretend that another face is the recognized one. During the truthful trials, it was their task to identify the known face and to pick any if no face seemed familiar to them. Lying was correlated with activation in the medial frontal gyrus, red nucleus, inferior frontal gyrus, supramarginal gyrus, superior frontal gyrus, dorsolateral prefrontal cortex (all occurring right-lateralized), and the bilateral precuneus.

As the entirety of the studies presented above clearly shows, we are not just dealing with one distinct network for face recognition but rather with a set of different brain areas that are more or less involved depending on the precise face processing demands. We thus have to face the far more difficult task of unraveling a multitude of interactions. While a complete understanding of face recognition on a neural level will probably elude us for quite a while, some neural correlates allow at least for a certain degree of predictability in a specialized setting.

## Memory and Face Processing in Offenders

Since we began to discuss the possibility of eyewitnesses operating as delinquents, we must be aware of the fact that offenders are eyewitnesses as well and that it is of high juridical interest to learn about their contingent memory specifics. However, whereas the mechanisms of face recognition are of particular interest in eyewitness testimony, they are not in the focus of attention in offender’s memory. Nonetheless, some insight could be gained referring to altered brain activation during face processing in offenders, for instance regarding emotional expressions as has been demonstrated in a couple of studies. In a case-report paradigm, Hoff et al. (2009) collected fMRI data from a psychopath with criminal background and a control group performing an *n*-back task with drawn facial expressions and scrambled drawings. The researchers found pronounced differences between groups: facial expressions involved phylogenetically older regions in the psychopathic participant, whereas only neocortical areas were activated in the control group. In another fMRI study, subjects labeled the sex of male and female faces with differing emotional expressions in varied intensities (Pardini and Phillips, 2010). Participants were chronically violent or non-violent men. The former showed reduced brain activation in the dorsomedial prefrontal cortex referring to faces in general, regardless of the expressed mood. In contrast, higher activation emerged in these regions with respect to mild fearful faces. The group of violent men also exhibited higher activation in the amygdala for neutral in comparison to happy faces. Very remarkable is the finding of an elevated activation for mild fearful faces in the violent group which could not be seen while watching neutral faces or faces with more pronounced fearful cues. The authors suggest that chronically violent men may interpret ambiguous facial expressions differently from others.

Criminals often exhibit mental disorders like addiction, amnesia, or psychopathy, and those characteristics could be also responsible for alternating results in memory or brain function (Parwatikar, 1990; Markowitsch and Staniloiu, 2011; Oszoy et al., 2013). In a word and face encoding fMRI experiment for example, alcohol-dependent patients did not exhibit the right-lateralized activation in the parahippocampal region during face encoding which had been observed in a healthy control group (Yoon et al., 2009). Concerning the immediate effects of alcohol on memory encoding, Söderlund et al. (2007) reported alcohol impaired memorization for object pairs and face-name pairs (but not for words and phrase-word pairs) that was associated with reduced bilateral prefrontal activation (cf. also the above-mentioned investigations on the state-dependency of memory under alcohol influence). Other differences between groups were found in the parahippocampal gyrus. In cases of antisocial personality disorder, discrepancies in brain function have been observed as well. In an EEG study by Pfabigan et al. (2012) with happy and angry faces as feedback stimuli, participants with antisocial personality traits displayed a smaller event-related P1 amplitude than participants with low antisocial personality scores.

Although face identification by offenders is of less juridical importance, comprehension of those distorted processes and influences on memory might lead to a better understanding of offender behavior. Usually, the more intriguing question in this context concerns the details of the crime, which may include the description of the victim but mostly highlight the act and circumstances of the perpetration. A deeper understanding of brain functions dealing with the representation, encoding, and recall of event memory would make an important contribution. Hasson et al. (2004) used functional brain imaging to demonstrate parallels in brain activation between subjects watching a movie. They found intersubject synchronization in multiple cortical regions. The results lead to the assumption that identical events might be processed in a similar way in different individuals. However, is this conferrable to criminal events? Offender and victim (to a certain extent) experience the same incident, but their particular role, their thoughts, and emotions will obviously be totally different from each other. While a crime in many cases is mostly traumatic for victims, possible occurrence of pleasurable feelings is also discussed in offenders (Evans, 2006). But although research normally focuses on trauma in victims, offenders can also suffer from intrusive memories related to their crimes and even develop a PTSD (Evans et al., 2007b). It is difficult to make a statement concerning prevalence, and indications vary in a wide range over studies (Evans, 2006). Pollock (1999) reported PTSD occurring in 52% of the 80 investigated perpetrators who were accused of committing homicide. Probability for developing PTSD depended on the offender’s personality traits and the form of violence chosen. Evans et al. (2007a,b) found in a sample of 105 offenders, who had been convicted of killing or seriously harming someone that 46% suffered from intrusive memories and 6% from PTSD. The authors assume that factors predicting reexperiencing symptoms like flashbacks in victims could be generalized to culprits. The probability of developing PTSD is also influenced by the impulsivity of a crime. Reactive homicide for example refers to a spontaneous and emotional driven aggression and yields a high risk of evolving negative feelings which may lead to PTSD. Instrumental homicide in contrast is goal-directed, planned, and proactive. There is no actual provocation required and the victim can be completely meaningless to the culprit (Christianson et al., 2007). On the other hand, not only unrequested memories like in PTSD can plague offenders, dissociative amnesia for the offense can occur as well. In a population of 207 convicts sentenced to life imprisonment, Pyszora et al. (2003) found amnesia primarily to be connected to preceding alcohol abuse, blackouts, psychiatric disorders, and crimes of passion. Among the 105 perpetrators studied by Evans et al. (2009a), 19% stated to have partial amnesia and 1% complete amnesia for their offense (cf. Parwatikar, 1990). The type of crime also affects the probability of developing amnesia. Reactive violent offends lead to memory loss more often than instrumental violent crimes (Cooper and Yuille, 2007).

Investigators do not only have to face several memory distortion phenomena while working with delinquents. They also have to calculate the risk of deceitful tendencies like denying or malingering. For a perpetrator, deceiving about what he or she did or at least feigning a memory impairment can be appealing in terms of some legal implications, like criminal responsibility and competency to stand trial (Porter et al., 2001). A culprit who cannot remember the details of his or her crime can hardly make an expedient contribution to his defense. Expert advice is needed in those cases to proof if the memory impairment is caused by organic disease, dissociative amnesia, a psychotic episode, or feigned amnesia (Bourget and Whitehurst, 2007), though a differentiation between dissociative (also called “psychogenic”) amnesia and amnesia with organic origin is questionable (Barbarotto et al., 1996). Markowitsch and colleagues (Markowitsch et al., 1997, 2000a; Markowitsch, 1999) reported deviated brain activity measured by PET and SPECT in several patients with dissociative amnesia diagnosis. Feigned amnesia is not only problematic because it has to be detected in the first place. It can also impair memory performance. After a mock crime, van Oorsouw and Merckelbach (2004) instructed a group of participants to feign amnesia in a free recall test. A week later, participants completed the free recall test again but were briefed to respond honestly. Their performance was compared to the results of two control groups: one group made honest efforts on both tests, the immediate and the delayed, the other group only attended on the delayed test. The group that had been honest all the time and took part in both trials outperformed the simulators and the controls that underwent the test for the first time. The authors suggest that malingering might have similar effects as a lack of rehearsal. Analogous results have been observed in other studies (Christianson and Bylin, 1999; van Oorsouw and Merckelbach, 2006).

This may also be very important in the light of offenders’ often claimed wish to forget about their crime. Next to a lack of rehearsal, several other reasons must be considered to possibly evoke amnesia (Christianson et al., 2007). For example, the differences between the highly emotional and arousing state during the act of crime and the calm environment of the criminal investigation could hinder correct retrospection, whereas recreating the context and the internal state during the crime could facilitate memorization (cf. the state-dependency of memory; Markowitsch and Staniloiu, 2012). Other conceivable explanations are intoxication, head injuries, brain diseases, or failures in meta-memory, i.e., for example the own conviction of being amnesic.

Several attempts have been made to detect lying using brain imaging technology, like in the above-mentioned study by Bhatt et al. (2009). And in pathological liars, white matter seems to be reduced in prefrontal regions (Yang et al., 2005). Markowitsch et al. (2000b) compared brain activation corresponding to real and fictitious autobiographical events and found the original events to evoke a neural response in the right amygdala, the right temporofrontal junction areas, and other cortical regions, while the invented stories led to activation in the precuneus. The differences may be caused by the stories’ unequal emotional attraction and the precuneus’ well-known role in mental imagery.

The question arises, whether neural correlates of delinquent behavior exist. Some evidence has been found concerning brain structure and brain activity alteration in individuals with criminal background or antisocial personality disorder. Differences can be observed, e.g., in the frontal lobes, in frontotemporal regions, and in limbic structures (Bassarath, 2001). But caution is indicated, since some of these changes can be found in persons not perpetrating crimes as well, and so in this context, no exclusive relationship between brain and behavior anomalies has been proofed to date (Markowitsch and Kalbe, 2007).

These findings illustrate that offenders’ memory abilities might differ in many ways from eyewitnesses’ capacities for remembering. The differences may be due to the perpetrators specific involvement in the act of crime, comorbidities like intoxication, PTSD, amnesia, or antisocial personality disorder or intended forgetting. A divergence in face processing is accompanied by a change in brain function and may also modulate emerging memory tracks for face stimuli even under normal conditions. Finally, the questionable truthfulness of the investigated persons exacerbates the researcher’s effort of shedding some light into culprits’ powers of recollection.

## Reality vs. Laboratory

Science has already made a wide range of contributions to legal implications concerning the treatment of eyewitnesses in recent years, like the cognitive interview (Holliday et al., 2012; Sharman and Powell, 2013) or several approaches of evolving facial composites (Frowd et al., 2012a,b). But in reforming the existing procedures, we have to weigh costs against benefits (Clark, 2012). In many cases, a reduction of false identifications is accompanied by a reduction in correct identifications as well. Further investigation is needed, and with the advent of neuroimaging techniques, research also starts to unravel the underlying neural mechanisms involved in person identification and face recognition. But these methods rely on laboratory settings and presumably lack ecological validity. While examining culprits, reliable data is even more difficult to obtain, as an offender’s cooperation without any intention of deceit cannot be taken for granted. Rightly the question arises whether results obtained under such laboratory conditions can be transferred to real felonious cases.

In the field of neuroimaging, original data of real-life events is obviously hard to come by. However, even if such precious pieces of data could be obtained, their interpretation would be more than challenging. Lacking the standardized methods used in experimental settings, performances of different eyewitnesses or offenders, perhaps even across different cases, would be rarely comparable. Nevertheless, the question arises whether laboratory studies can teach us something about the nature of eyewitness memory and what pitfalls might be waiting in their analysis.

A compromise is attempted in so-called ecologically valid testing situations. One such study was performed by Mohamed et al. (2006) who tested normal individuals under two opposing conditions. In one the subjects agreed to the statement that he or she fired a gun, and in the other they disagreed. In this way the same individual could be tested with functional brain imaging under both the lie and the truth condition. As expected from the results of subsequent studies (e.g., Markowitsch, 2008; Spence and Kaylor-Hughes, 2008; Markowitsch and Merkel, 2011), there was more activation during the lie than during the truth condition. Frontal, temporal, cingulate, fusiform, insular, and occipital areas were activated during the lie condition, while for the truth condition there was more limited frontal and temporal activation, possibly including the lenticular nucleus.

Ihlebæk et al. (2003) compared eyewitness memory originating from a video and a live condition. Subjects in the live condition took part in a staged bank or service station robbery. Robbers were performed by two police officers. For the video condition a recording was used which was made by one of the researchers. Their major finding was the difference between groups concerning the number of details reported, with participants in the video condition outmatching the other subjects. They reported more details and were more accurate. Nevertheless, the patterns of mistakes were quite similar, for example both groups over- or underestimated event duration and age of the robbers. As the authors point out, the lower rate of memorized details in the “live” group might be due to the fact that witnesses may have had less opportunity to watch the robbers closely, since some of them have been forced to get down to the floor and to cover their faces. This is supported by the fact that a high proportion of the differences between groups can be explained by “I don’t know” answers. The authors outline that laboratory experiments may overestimate eyewitness memory, but that the kind of errors that are made are quite similar in both settings. It can be argued that a staged robbery still is an artificial event, in particular because the participants have been informed.

Wagstaff et al. (2003) conducted two studies to analyze archival testimonies of 70 and 48 real crime witnesses, respectively. In all cases the particular culprit was arrested and convicted. They tried to discover, how the level of violence, the presence of a weapon, and the age of witnesses affect memory accuracy regarding the offender’s age, height, build, hair color, and hairstyle. Violence (and also partly the type of crime) predicted memory performance concerning hair color; the higher the level of violence, the more accurate the victims’ judgments regarding this aspect. Witnesses to rape also gave more precise statements concerning hair color than witnesses to robbery. But both factors, violence and type of crime, were not unrelated, since crimes of rape involved higher rates of violence in the investigated cases. Closer distance to the culprit, a longer exposure time (Memon et al., 2003b), or the higher probability of knowing the offender are conceivable explanations which might have contributed to these results.

**Figure 1:**
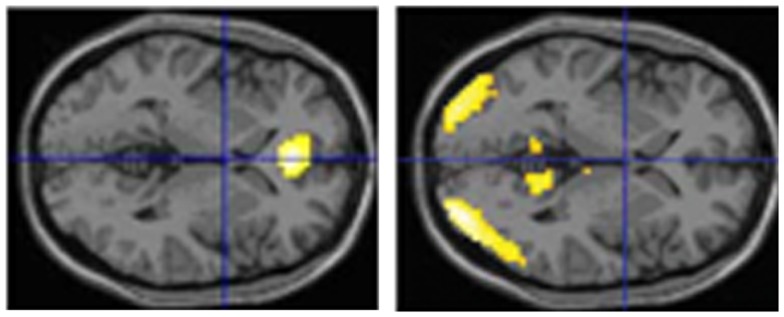
**Brain imaging activations during correct or false identification of visual stimuli in the study of Kühnel et al. (2008) (horizontal sections; left: activations during correct answers, right: activation during false answers)**.

We have performed two experiments in which we tested eyewitnesses under laboratory conditions. In the first study (Kühnel et al., 2008), normal participants studied short movies (each of less than 4 min duration). Thereafter they had to respond with YES (seen) or NO (not seen) to individual pictures which either stemmed from the movie or not. Some of these single shots had a high likeliness of having been in the respective movie and some not. Overall, participants made almost 45% erroneous responses. However, brain images obtained with functional magnetic resonance imaging (fMRI) revealed a more clear-cut picture: the correctly identified pictures led to a medial prefrontal/anterior cingulate activation while the falsely identified resulted in activations in the visual association cortex and the precuneus (all bilaterally) (Figure 1). In a second study (Risius et al., 2013) we again used a film and asked normal participants later during fMRI to judge whether a statement referring to the film was correct or not. Furthermore, they had to give confidence judgments for each choice and – if they wished – they could bet that their answer was correct. If this happened, activations were found in temporal, frontal, and middle and posterior cingulate areas as well as in the precuneus. Otherwise cingulate and medial temporal regions were activated. Withholding an answer (as compared to volunteering it) resulted in increased bilateral hippocampal activations as well as in an activation in the left caudate nucleus.

Several factors may influence eyewitness memory and especially by the use of field studies it is difficult to discover which of them are crucial and how they might interact with each other. Laboratory experiments can manipulate single factors and give us an idea of the underlying processes, restricted to the fact that experimental designs differ in many characteristics from real criminal experiences. But even those authentic events differ in many aspects such as stress-level, amount of violence, weapon presence, distance to the offender, and if he or she is familiar to the eyewitness. Many attempts have to be made to complete this mosaic. Therefore Chae (2010, p. 259) concluded: “No single study, either naturalistic or experimental, can cover all the relevant factors present in forensic situations.” With regards to the benefit of field tests of eyewitness identification procedures Schacter et al. (2008, p. 5) recommended: “No single field study can produce a final blueprint for procedural reform; we will need many.” And so both approaches – field and laboratory research – bring their advantages into account: ecological validity and experimental control. But a lot of effort has to be made to compensate for their disadvantages and to integrate the findings into a coherent picture.

## Conclusion and Outlook

The multitude of parameters affecting eyewitness’ and offender’s memories illustrates the need for standardized methods regarding the treatment of witnesses and suspects. But not only police officers have to be sensitized for the influences their interview techniques might have on the reliability of testimonies and how best recollection results can be obtained. Also judges, advocates, and jurors must be informed about the differences between procedures, the impact they have on memory quality and other factors working in favor of memory distortion. It is science’s responsibility not only to evolve, develop, and test those methods in cooperation with operators, but to educate judging persons how results have to be interpreted and how much value should be attached to them (Markowitsch and Merkel, 2011; Markowitsch and Staniloiu, 2011; Schacter and Loftus, 2013).

This is also true for the upcoming neuroimaging techniques like fMRI. The investigation of brain function in criminal contexts is a young field of research. The prospect of detecting false memories in eyewitnesses and deception in offenders is highly appealing. Although, achievement of these objectives would render a great service, it is still quite a long way before this technique is able to clarify ongoing brain processes in a reliable manner. Neuroimaging data is hard to interpret; all the more because we lack ecologically valid studies. Face processing for example is modulated by facial characteristics and how familiar the shown person is to us, but our feelings for this person will affect our brain activity as well. It does make a difference if we watch a beloved or a neutral person’s face or if we look at someone we might fear or be angry about. Encouraging are the results of Lefebvre et al. (2007) who found the event-related brain potential P300 to indicate correct target identification in a lineup task. Nevertheless, it still has to be explored to what extent these findings can be transferred to other criminal contexts.

Investigation of perpetrators’ memory is even more demanding, since results are often confounded by different comorbidities, like intoxication or personality disorders. Those concomitant phenomena can cause alternated face processing even under normal conditions. If we want to learn about offenders’ brain functions, we must examine those processes in different settings and untangle the confounding factors, but we also must control for deceitful tendencies culprits might have.

Accordingly, we have to meet the challenge of taming the technical demands on the one side and to discriminate the neural activity associated with a special mental state on the other. We still have to learn a lot about brain function in the context of criminal events to know what we are searching for in an eyewitness’ or offender’s head. Even if we could rely on authentic data, fMRI technique still has to be improved. Up to now it is not possible to reliably detect false memory or deception by brain imaging outside the laboratory. Laymen might believe in data obtained by brain scans as objective proof of ongoing brain function, but it has to be pointed out that it is a product of many decisions made by the researcher, like the extent of the statistical power, conformation of individual brain structure to a standardized anatomical brain model, and other fine adjustments and corrections (Bumann, 2010). Furthermore, it is dangerous to draw conclusions from group analysis to single subjects. Group data are achieved by averaging data across individuals. In the extreme, it is possible that no single subject exhibits an activation pattern as the average mean suggests. Another risk consists in making a wrong deduction by “reverse inference” (Poldrack, 2006), that is to infer the presence of a special mental state or function on the finding of a special brain activation pattern. If such a pattern of activity is found, we cannot be sure that a special mental state, which also has been observed to show this pattern, is indeed present, since brain structures normally fulfill many different tasks. On the other hand, the absence of a pattern, correlated to a special mental state in group analysis, does not mean that the state of interest must be absent as well, as other brain areas could execute the respective cognitive function. Nevertheless, the potential of these techniques is enormous, and there is a lot to learn on the way. Therefore, it is important not to lose courage, to make the attempt to unravel the underlying processes, and to obtain data from real cases.

## Conflict of Interest Statement

The authors declare that the research was conducted in the absence of any commercial or financial relationships that could be construed as a potential conflict of interest.
